# Pomegranate: A review of the heavenly healer’s past, present, and future

**DOI:** 10.22038/IJBMS.2023.72816.15844

**Published:** 2023

**Authors:** Mehran Mohammadi, Zahra Boghrati, Seyed Ahmad Emami, Maryam Akaberi

**Affiliations:** 1 Department of Pharmacognosy, School of Pharmacy, Mashhad University of Medical Sciences, Mashhad, Iran; 2 Department of Traditional Pharmacy, Mashhad University of Medical Sciences, Mashhad, Iran

**Keywords:** Ellagic acid, Ethnopharmacology, Persian traditional medicine, Pomegranate, Punica granatum

## Abstract

In the great Persian Empire, pomegranate (*Punica granatum* L.) had a wide reputation for use both as an herbal medicine and nutritious food. It was also a symbol of peace and love according to Achaemenid limestones in the great Persia. This paper aims to review the traditional uses of pomegranate in Persian and Islamic traditional medicine and have thorough and current information regarding the pharmacology and phytochemistry of this valuable plant for practical use and further research. Relevant information about *P. granatum* was collected from scientific publishers and databases including Elsevier, Wiley, PubMed, and Google Scholar between 1950 and 2022. The traditional knowledge was extracted from Persian and Islamic traditional textbooks. Based on traditional textbooks, pomegranate has beneficial effects on diseases related to gastrointestinal, upper and lower respiratory, visual, and reproductive systems. In addition, pomegranate and its preparations have been prescribed for treating metabolic disorders, skin problems, and wounds as well as dental protection. Preclinical and clinical evidence supports many therapeutic potentials of pomegranate in traditional medicine. Its therapeutic effects are mostly attributed to its polyphenols. The knowledge in Persian and Islamic traditional textbooks about pomegranate and its preparations can be used as a guide for further preclinical and mainly clinical studies to discover the therapeutic potential of this valuable plant.

## Introduction

**Figure 1:**
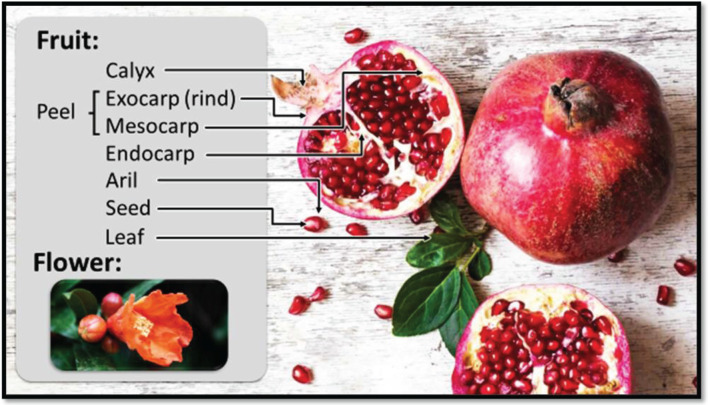
Different parts of *Punica granatum* (©Nutritionfacts.org)

Pomegranate (*Punica granatum* L.), belonging to the *Lythraceae *family, is a historic fruit that is indigenous to Central Asia and may be found in places like the Middle East, Iran, and Turkmenistan to northern India (1). *P. granatum* (Figure 1) is a fruit-bearing shrub or a small tree that grows up to 501507 m with very diverse varieties (2). Pomegranate and its components were found to have powerful anti-oxidant, anti-inflammatory, antifungal, antibacterial, and antimicrobial effects, according to studies conducted in both *in vitro* and *in vivo* over the past few decades (1). In addition, some animal studies have shown that pomegranate may have anti-hypertensive and antiproliferative properties (3). Furthermore, pomegranate juice or extracts have been shown in multiple pre-clinical and clinical trials to have positive benefits on a number of disorders, including respiratory diseases (4), digestive problems (5), neurodegenerative diseases (6, 7), metabolic disorders (8, 9), cancer (3, 10), osteoarthritis (11), skin problems (11), etc.

Traditionally, pomegranate and its products have been used for the treatment of several health problems. In Islamic and Iranian Traditional Medicines (ITM), this valuable plant was used by traditional physicians in various preparations and diverse application forms for a wide variety of illnesses. They used different parts of the plant mainly fruit peels, fruit juice, and flowers for health problems such as skin diseases, reproductive problems, gastrointestinal disorders, infectious diseases, and respiratory problems (12-19). As mentioned by ITM physicians, most of the beneficial properties of pomegranate are due to its astringent effect. Modern medicine relates this astringency to the presence of phenolic compounds including tannins. Many available pomegranate formulations in the market have been prepared according to traditional knowledge. For instance, anti-aging and cosmetic products from pomegranate are available in the market under many brand names. 

Although pharmacological and clinical studies have confirmed several therapeutic effects of pomegranate products and pomegranate constituents, by looking into the uses of this plant in traditional texts, many applications can still be extracted. Nevertheless, there are still many potential applications in the traditional references that can be useful for possible formulations. To the best of our knowledge, there is no similar article discussing the traditional uses of pomegranates in Islamic and Persian medicine. Thus, in the current study, we aimed to have a comprehensive review of the application of pomegranate in ITM and compare it with modern medicine. A recent example of overlapping the traditional and modern uses of this plant is Covid-19. ITM physicians prescribed pomegranate for cough and cold, and as an antimicrobial agent. Interestingly, according to research, *P. granatum* and the polyphenolic components it contains may be useful against Covid-19 (20).

***Phytochemistry***

Depending on the cultivar, growing area, maturity, cultivation technique, climate, and storage conditions, different portions of the pomegranate have distinct chemical compositions. Although in each part of the plant, a group of specialized metabolites may dominate, the most important and biologically active constituents are polyphenols and tannins. For instance, pomegranate polyphenols particularly the anthocyanins might have significant effects against metabolic syndrome (21). Nevertheless, pomegranate includes several kinds of phytochemicals, including flavonoids, alkaloids, both condensed tannins (proanthocyanidins, are polymers formed by the condensation of flavans), and hydrolyzable tannins (ellagitannins and gallotannins) and organic acids (Table 1) (22).

*Polyphenols*

Pomegranate is a rich source of polyphenols including phenolic acids, anthocyanins, and hydrolyzable tannins. Several hyphenated analytical methods such as Ultra high-performance liquid chromatography-mass spectrometry (UHPLC-MS^n^) have been used to examine the polyphenol profiles of different pomegranate sections (23, 24).

*Ellagitannins*

Ellagitannins are polymeric compounds that frequently have various amounts of galloyl and hexahydroxydiphenoyl units attached to glucose. One of the metabolite categories in pomegranate that has been the subject of much research is ellagitannins. These substances, which are converted to urolithins in the digestive system, are the primary bioactive phytochemicals in pomegranate juice (22). Ellagitannins vary from gallotannins in that their galloyl groups are connected by C-C bonds. Additionally, gallotannins do not often form macrocycles, but ellagitannins do (25).

*Gallotannins*

Pomegranate contains some gallotannins. Gallotannins are a variant of hydrolyzable tannins. Gallotannins are polymers created when the hydroxyl group of a polyol carbohydrate, such as glucose, esterifies and bonds with gallic acid, a polyphenol monomer. For instance, 1,3,4-trigalloylglucose may be found in the leaves of pomegranates. Weak acids or bases hydrolyze this type of tannin to create glucose and phenolic acids (22, 25).

*Lignans*

Pomegranate has been shown to contain a variety of lignans and phenylpropanoidic metabolites with estrogenic action (22, 26, 27).

*Phenolic acid derivatives*

Phenolic acids including chlorogenic, caffeic, syringic, sinapic, *p*-coumaric, ferulic, vanillic, ellagic, gallic, and cinnamic acids have been identified in pomegranate peel (22, 28).

*Flavonoids*

Flavonoids belonging to different subclasses including, flavan-3-ols like (+)-catechin, (−)-epicatechin, and (+)-gallocatechin, flavonols, flavanones, dihydrochalcones, and flavones have been identified in pomegranate (22).

*Organic acids*

Citric and malic acids are among the organic acids identified from different parts of pomegranate (22).

*Anthocyanins*

Pomegranate juice’s crimson hue is a result of anthocyanins. Pomegranate anthocyanins such as cyanidin, pelargonidin, and delphinidin have been found to be conjugated with one or two hexose sugars (22).

*Alkaloids*

The three main alkaloids found in the stem and root barks of pomegranates are pelletierine, pseudopelletierine, and N-methylpelletierine (29). Sedridine, 2-(2′-hydroxypropyl)-∆1piperideine,2-(2′-propenyl)-∆1piperideine, norpseudopelletierine, and the pyrrolidine alkaloids (with a five-membered N-containing ring) hygrine and norhygrine have also been found in root barks of pomegranate at small amounts. In addition to the alkaloids that build up in root and stem barks of the plant, N-(2′,5′-dihydroxyphenyl) pyridinium chloride has been identified in pomegranate leaves, and a pyrrolidine-type alkaloid punigratane (2,5-diheptyl-N-methylpyrrolidine) has been recently characterized in pomegranate fruit peel (22).

***Pomegranate in Iran and ancient Persia***

**Figure 2:**
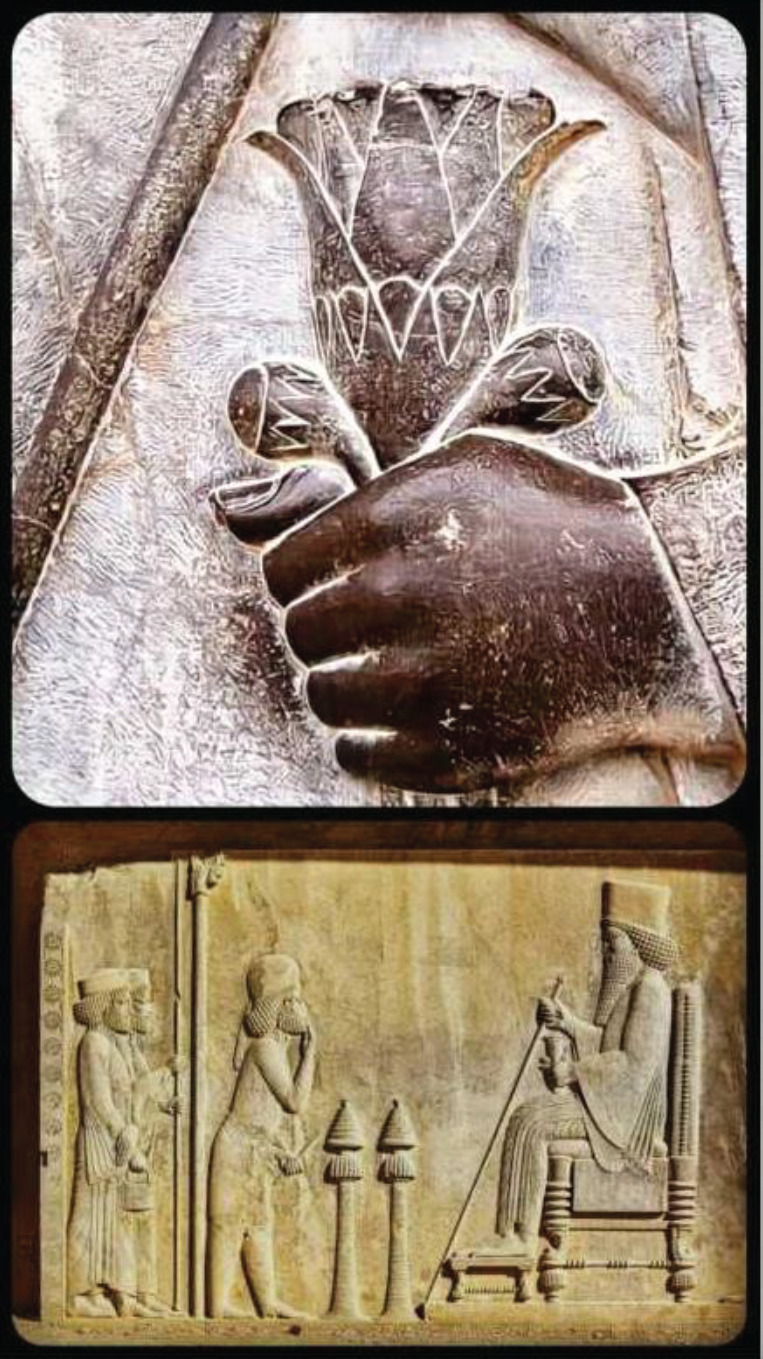
A Persian Achaemenid limestone bas-relief. (©Memranet.com)

**Figure 3:**
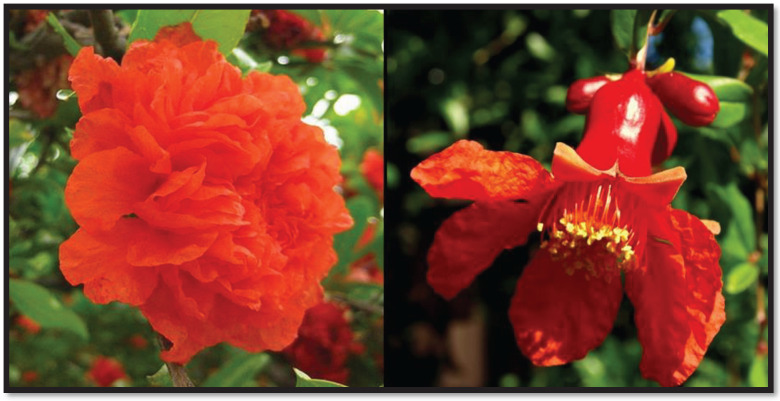
Blossoms of *Punica granatum*

Pomegranate production and exports from Iran rank among the highest in the world (71). Pomegranate, known as “Anar” in Persian, grows widely and is cultivated throughout Iran. As a common food, Iranians use pomegranate fruit juice and paste in many dishes including sour chicken, Fesenjan, and Lavashak. Iranian culture has long used the pomegranate as a symbol. For instance, Isfandiyar, a legendary Persian warrior, gains invincibility by eating a pomegranate. The Persian phalanx’s spears were decorated with golden pomegranates, according to Herodotus’ “The Persian War.” (72). Interestingly, the blossom of pomegranate (Golnar and Gole-e-anar) is the symbol of peace, love, and kindness in Iranian culture. In a Persian Achaemenid limestone bas-relief (Figure 2), a flower can be seen in the hand of the king (maybe Darius I or Xerxes) as a symbol of peace. It is interesting to note that the blossom of *P. granatum* var. *pleniflora* (Golnar-e Farsi) is known as Golnar and the flowers of other varieties are called Gole-e-anar. Persian Golnar is a plant that is grown for ornamentation and produces unproductive blossoms (Figure 3).

***Pomegranate in Islamic Traditional Medicine (ITM)***

Two kinds of pomegranates are described in ITM textbooks namely wild and cultivated ones with two distinct tastes sweet and sour (12). However, some ITM scientists believed that there are four tastes: astringent (un-ripped pomegranate), sweet, sour, and sour-sweet. Pomegranate’s temperament differs by its type; the sweet one is cold and moist in the first degree, and the sour type is cold and dry in the second degree. The sour-sweet pomegranates, tend to be moderately dry and cold (for more information about the humors you can see Bone and Mills (73)). Sweet pomegranate is good for cold people because it makes their stomachs warm, but harmful for people with acute fever. Sour-sweet pomegranates are good for people with moderate temperament.

From a traditional point of view, pomegranate, as a low-calorie fruit, is generally an astringent, drying, and cooling agent. The most reported medicinal activities for pomegranate are attributed to its astringent property that stimulates the contraction of bodily tissues; often used to soothe the skin and stop bleeding. All types of pomegranates are astringent, however, depending on the types (sweet, sour, and sour-sweet), the benefit of each is according to the prevailing taste (14, 19). It is believed that the astringency and drying properties of the pomegranate seeds are more than the juice and of the peel more than both and interestingly of the un-blossomed flowers (that fall from the trees) much more than all the latter (19). The pomegranate seed is more drying than its juice, but the peel and fleshy mesocarp are more drying than the seed. The drying power of the flower of pomegranate (known as Golnar in Persian) is like peel and fleshy mesocarp. Golnar is used as a highly astringent agent in many traditional prescriptions such as treatment of the wounds (14, 17, 19). Moreover, the astringency property of all parts of sour pomegranate is more than the sweet ones (19, 74). A point that should be considered is that when talking about the seeds of pomegranate (known as Nardoon in Persian) in ITM, it means the sun-dried seeds with the arils not the seeds as a waste or byproduct (12-15, 19, 74-76).

Interestingly, almost all parts of the pomegranate, including, fruits, seeds, peel, leaves, bark, root, flowers (ripped and un-ripped), and even the calyx and stamens (pomegranate’s crown) are used medicinally in ITM. In the following paragraphs, all the mentioned medicinal applications of pomegranate in major ITM textbooks will be discussed briefly and its applications in modern medicine will be cited in detail.

*Gastrointestinal system*

Pomegranate is believed to be useful for stomach and intestines (17, 18, 74). In some ITM textbooks, it is mentioned that while sweet pomegranate has beneficial effects on the digestive system, sour pomegranate is harmful to the stomach and scrapes the bowels. Therefore, it should be eaten with sweeteners like Halva Ardeh (a kind of Iranian sweet made of sesame and sugar) or honey (13, 15). Avicenna also believed that while the sweet and sour-sweet pomegranates are useful for the stomach, the sour one is not good. He emphasized that the benefits of sweet pomegranate for the stomach are even more than apples and quince (17). In Menhaj: if old people want to eat a sour pomegranate, they should mix it with Balang jam (a kind of jam made from the peel of *Citrus medica*) (15). Al-Aghraz: both sweet and sour pomegranates are described to remove stomach heat; however sweet pomegranate is useful for the stomach and sour one is harmful (18). On the contrary, in many ITM textbooks such as Al-Jamee, sour pomegranate is also described to have beneficial effects for inflamed stomachs (14). Al-Jamee: both sour and sweet pomegranates if extracted along with their fleshy mesocarp and mixed with red sugar are stomach-strengthening agents (14). The probable point is that in Al-Jamee, sour pomegranate is prescribed with its mesocarp which has astringent properties, so it would tan the stomach. Also, Hakim Mohammad Momen Tonekaboni believed that the extract of both sweet and sour pomegranates along with their fleshy mesocarp would strengthen the stomach (75).

Razi believed that if the seeds of sour pomegranate (sun-dried seeds) are used in food, it would prevent the flow of excess humors to the stomach and intestines (14, 19). And elsewhere in his book, it is said that pomegranate juice has the property of preventing the flow of wastes into the stomach and intestines. It is also useful for treating a fever that causes diarrhea.

In many of the studied references, an anti-parasitic activity is described for pomegranate (18, 75). Razi in his book Al-Hawi has mentioned: “Pomegranate root skin when cooked with rock candy is useful for treating abdominal worms and removing Taenia eggs” (19). Ibn Beytar in his book Al-Jamee Le-Mofradaat al- Adwiah wal- Aghḏiyah (Comprehensive book in Simple Drugs and Foods): Administration of pomegranate peel in patients with intestinal worms and drinking high-temperature water after that would remove the parasites (14). Hakim Mohammad Momen Tonekaboni also believed that the administration of a drink prepared from the milled pomegranate skin in warm water is a certain cure for patients with intestinal worms (14, 75).

In many ITM textbooks, the beneficial properties of pomegranate for healing intestinal wounds, its anti-diarrhea, and its stomach-strengthening activities are mentioned. It has been written in Al-Hawi that edible use of pomegranate calyx, tends to tan the stomach and dry digestive wounds (19). Purging an infusion prepared from pomegranate in combination with *Oryza sativa* (rice) and barley to the digestive tract has been prescribed for the treatment of diarrhea and intestinal wounds (14, 75). Sitting in a bowl containing an infusion of pomegranate is also recommended for healing intestinal wounds, particularly wounds in the lower parts of the intestines (19). Golnar is also mentioned to be useful for treating these kinds of wounds. Pomegranate seed extract, especially sour pomegranate, has been used for anal wounds and hemorrhoids when cooked with honey (19). If a peeled sour pomegranate along with its mesocarp is milled in a stone mortar and squeezed, then half of the obtained extract is mixed with 10 parts of red sugar and applied, it might cause laxative effects (15). The oral use of sour pomegranate is useful for bile diarrhea and nausea (19). Roasted pomegranate seed flour is a stomach-strengthening and anti-diarrhea agent (19). Pomegranate paste also strengthens the stomach by tanning it (19). Administration of 17-25 pills (pepper seed size) prepared from the skin of sour pomegranate cooked with concentrated vinegar would heal diarrhea, and stomach, intestinal, and anal wounds (14, 75). Administration of a mixture prepared of three to seven unripe pomegranate buds, some acacia leaf, and a little white cumin (milled in a stone mortar) for three to seven consecutive days would heal infants and children with diarrhea (the number of pomegranate buds depends on the child’s temperament, age, and physical strength) (13). In addition, preparation was made from the flowers of pomegranate for the improvement of hernia (19).

*Precautions*

The sweet pomegranate produces little heat and flatus so it is not recommended for people with a warm temperament like people with acute fever (19). Razi in his book (Eliminating the Harms of Foods): “Sweet pomegranates cause a bit of bloating. Eating after a meal will lower the food from the stomach and need no adjustment because the bloating will quickly subside. But sour pomegranates have a longer stopping time in the stomach, causing bloating and severely cooling the liver, especially if used continuously. It is more harmful to cold people because it cools their liver and prevents the liver from absorbing food and thereby causes diarrhea”.

*In other countries*

Chinese and Mexican populations have historically used pomegranate exocarp to cure gastrointestinal conditions like diarrhea, dysentery, and stomachaches (77, 78). The antidiarrheal activities of punicalagin, corilagin, and ellagic acid (found in ethyl acetate fraction) alone or in combination have been confirmed (78). In India, fruit juice is traditionally used to treat dysentery by mixing it with warm water twice daily and giving it to anemic people as a tonic. Ash produced by burning seeds has a styptic quality. Fruits are consumed in their natural form to treat jaundice and strengthen the heart. Bark powder is used as an astringent with a spoonful (79). Moreover, fruit is a good source of iron in Pakistan. The fruit of the plant is consumed to treat iron deficiency. Its bark is used to treat nasal congestion. When the fruit’s epicarp is dried, it is administered to cattle to cure diarrhea (80). Traditional Thai herbal medicine for treating diarrhea or bloody mucous diarrhea contains pomegranate pericarp. Studies were conducted on the formulation’s antibacterial activity against microorganisms known to cause diarrhea, such as *Staphylococcus aureus*, *Vibrio cholera*, and *Vibrio parahaemolyticus*. Except for *V. cholera*, all of the bacterial pathogens examined demonstrated inhibitory zones (6.3-14.8 mm) in response to the *P. granatum* extracts (ethanol and water)(81).

*Evidence from modern medicine (Gastrointestinal system)*

Decoctions and extracts from different parts of *P. granatum* fruit have been shown to alleviate gastrointestinal diseases such as diarrhea, gastrointestinal tumors, gastrointestinal infections such as infections caused by *Helicobacter pylori*, and inflammatory disorders. The studies consisted of *in vitro*, *in vivo*, and clinical studies (Table 2). Despite promising pharmacological activities observed in preclinical studies, there are still very few human clinical trials that study these effects. Hence, to thoroughly explore the benefits of pomegranate derivatives on the human gastrointestinal system and their safety, further clinical trials need to be done.

*Respiratory system*

Sweet pomegranate is useful for chronic coughing, the roughness of the throat, and chest pain and acts like a mucus-softening agent (12, 14-16, 19, 76). It is mentioned in Al-Hawi that when soaked in alum and rainwater, pomegranate is useful for the throat and lungs (19). Also, Avicenna believed when pomegranate seeds are mixed with rain water, it would be beneficial for the lower respiratory system (17). Studying the Islamic traditional textbooks showed that pomegranate is highly recommended for dry coughing (16). For instance, Ibn Beytar prescribed a mixture of pomegranate with the oil of sweet violet (*Viola odorata*) for dry cough (14). This beneficial effect is also mentioned by many other ITM physicians like Ibn Nafis Qarshi (16), Dawoud Antaki (12), and Ghasani (76). On the other hand, sour pomegranate is harmful to the respiratory system and it hurts the lungs and throat (15).

There is a tried prescription in several ITM textbooks for the treatment of chronic and dry coughing: “It would be a certain cure for chest pain and coughing when the pomegranate’s head is pierced and repeatedly filled with swe*et al*mond or viola oil (to the extent its capacity allows), then put on the fire to absorb the oil; drinking the obtained extract would completely remove coughing”. It is also mentioned that drinking PJ with sugar, starch, Arabic gum, and almond oil has the same effect (13, 14, 74, 75).

*Evidence from modern medicine (respiratory system)*

Different preparations from *P. granatum* and their major components such as EGCG have been reported to reduce the severity of respiratory system problems (Table 3). Some studies have evaluated the efficacy of* P. granatum* during the recent pandemic (101). In recent years, *P. granatum *fruit has been used to treat and prevent a variety of respiratory illnesses. Pomegranate fruit, juice, extract, peel powder, and oil have been shown in *in vitro* and *in vivo* studies to have positive effects on a variety of respiratory conditions, including asthma, lung fibrosis, lung cancer, chronic obstructive pulmonary disease (COPD), and alveolar inflammation by modulating a number of different mechanisms, including anti-proliferative, anti-oxidant, anti-microbial, anti-viral, anti-inflammatory, anti-cancer, and anti-tumorigenic effects. Nevertheless, pomegranate has been used in a limited number of clinical trials as an intervention for various respiratory disorders (4). Consequently, to confirm the efficacy of this natural fruit for the prevention and treatment of lung-related disorders, either alone or in combination with other medicines, well-designed human clinical studies are advised.

*Skin and wound healing properties*

One of the most mentioned properties of pomegranates in ITM textbooks is their wound-healing activity (12). Since the flowers, buds, and calyx of pomegranates are the most astringent parts, administration of them has been strongly recommended for the treatment of wounds and injuries, as well as removing scars. However, other pomegranate parts and preparations such as its juice and peels have also been used for this purpose. Razi in his book Al-Hawi has mentioned: “Due to its highly astringent, drying and cooling properties, Golnar when applied to wounds or scratches, quickly heals ulcers and blocks bleeding” (19). Administration of an ointment prepared by grinding pomegranate flowers or buds and mixing them with honey on smallpox scars and other wounds for several consecutive days would eliminate the scars (14). In many textbooks, the burned flower or calyx has been mentioned to have such activity (15-17). Moreover, sweet pomegranate extract benefits wounds and infections when cooked in a copper dish (14).

Pomegranate has also been prescribed for other skin problems such as paronychia, scabies, thrush, erysipelas, and pruritus (12-14, 16, 75). For instance, both sour and sweet pomegranate juice along with their fleshy mesocarp concentrated in a copper pot have been prescribed for treating paronychia and scabies (75).

*Evidence from modern medicine (Skin and wound)*

Pomegranate is known to minimize photoaging and chronological skin aging by different anti-oxidant and anti-inflammatory mechanisms. These effects are mainly due to the presence of potent polyphenols (ellagitannins and ellagic acid) (115). Pomegranate also has presented four effects considered important to the treatment of skin and soft tissue infections (antimicrobial, anti-oxidant, anti‐inflammatory, and healing) (116). Pomegranate whole fruit is considered to have protective activity against chemical-induced and ultraviolet (UV) radiation-mediated cutaneous damage, including carcinogenesis (117).

The studies that have been done on this topic consist of *in vitro*, *in vivo*, and clinical studies (Table 4). These studies mostly show anti-aging, UV radiation protection, wound healing, and skin-lightening effects of pomegranate extracts. Most of the clinical studies have been conducted on the anti-aging effect of this plant’s extracts, and they concluded that pomegranate extracts can alleviate the skin aging process in humans.

*Reproductive system*

It is believed that sour pomegranate because of its cold and dry temperament may decrease the libido and the production of semen so it is better to be served with agents with a hot temperament like ginger jam, strong wine, and Shorba (a kind of soup containing garlic and aromatic spices)(74, 75).

Administration of pomegranate preparations such as its juice and paste has been repeatedly prescribed in ITM for decreasing nausea during pregnancy. Tonekaboni has mentioned in his book Tohfah: “Eating the flour obtained from dried seeds of pomegranate is useful for pregnant women who are willing to eat soil and mud” (75).

Pomegranate is believed to be useful for treating uterine wounds, infections, and inflammations (14, 19). Ibn Beytar has written in Al-Jamee: “Using a purged extract obtained from boiled PJ in combination with dill would heal the chronic infections in the uterine (14). He also added that sitting in a decoction from pomegranate peels has the same activity (14). And Razi said in Al-Hawi: “Sitting in a decoction prepared from the seeds of sour pomegranate is useful for the treatment of infections and inflammations in women’s reproductive system” (19). There is also a similar healing property for the flowers of pomegranate (Golnar) (19).

Pomegranate peel, due to its astringency and drying properties, benefits women with extra bleeding in their menstrual period. Tonekaboni prescribed sitting in a decoction of pomegranate peels in these circumstances (75).

*Evidence from modern medicine*

One of the main mechanisms of action for pomegranate against diseases related to the reproductive system is anti-oxidant activity. Age-related sexual dysfunctions are exacerbated by etiological variables such as organ damage, degenerative illnesses, and the strains of modern life. According to studies, pomegranate can reduce ROS activity in the testis and other organs. Sperms’ plasma membrane is mostly composed of unsaturated fatty acids. It is hence especially vulnerable to oxidative damage. Spermatozoa membranes’ lipid matrix is destroyed by lipid peroxidation, which is also linked to impairments in membrane integrity and loss of motility (139). Thus, pomegranate and its derivatives can inhibit free radicals via their anti-oxidant activities (140), improve sexual dysfunctions, and ameliorate oxidative stress and aging-induced related damages (Table 5). According to Table 5, most of these studies were *in vivo* and there is a need for more clinical studies to survey the effects of *P. granatum* and its derivatives on the reproductive system.

*Mouth and teeth*

Again, because of its drying and astringent properties, pomegranate (particularly its flowers) has had traditional applications for mouth and teeth problems such as repairing loose teeth, oral ulcers, toothache, gum bleeding, and improving gum health (14, 19, 75, 76). A mouthwash from Golnar has been used to stop bleeding gums and repair loose teeth (19, 75). Heravi has prescribed Golnar for the treatment of mouth ulcers and infections as well as bad breath (74). Tonekaboni even suggested PJ for the treatment of malignant mouth ulcers (75). Sour pomegranate extract is useful for infectious mouth ulcers (14). Pomegranate seed extract, especially sour pomegranate, has been used for oral wounds when cooked with honey (19). Keeping PJ in the mouth has been used to strengthen the gums and heal malignant mouth ulcers (75). Interestingly, Heravi believed that pomegranate blunts the tooth because of its astringent properties, and added that sour pomegranate destroys the tooth (74).

*Evidence from modern medicine (mouth and teeth)*

The efficacy of pomegranate has been evaluated for the treatment of mouth and teeth problems. The studies show that pomegranate is effective for a range of mouth and teeth diseases such as aphthae and chronic periodontitis (149). Mouthwash containing pomegranate extract may help prevent dental plaque and tartar buildup by stifling the activity of the bacteria that produce plaque and reducing their capacity to cling to the tooth structure. In addition to reducing oxidative stress in the oral cavity, flavonoids, one of the active components of pomegranates, are thought to prevent gingivitis through a variety of other processes (150).

The anti-microbial activities of pomegranate have been confirmed by several *in vitro*, animal, and clinical studies. For instance, in a study, pomegranate has shown interferences with biofilm formation against antibiotic-resistant bacterial strains and quorum sensing among biofilm microorganisms (151). Studies have revealed that pomegranate is effective against a wide range of oral pathogens including, *Streptococcus salivarius, S. sanguis, S. mitis, Porphyromonas gingivalis, Aggregatibacter actinomycetemcomitans*, and* Prevotella intermedia* (151).

In a study on the effectiveness of *Achyranthes aspera*, 0.2% aqueous chlorhexidine gluconate, and *P. granatum* mouthwashes on salivary *S. mutans* count in children, chlorhexidine showed better results than the other two. However, all showed a statistically significant reduction of *S. mutans* count and plaque index after 7 days (152). However, in another RCT on thirty children aged 6-12 years old, the activity of PPE was investigated against this pathogen and no statistically significant difference was observed between PPE and chlorhexidine as a positive control (Table 6).

*Eyes, ears, and nose*

Administration of a mixture of concentrated PJ, particularly sour pomegranate, with honey has been used for earache and deep nasal wounds (19, 75). Ibn-e-Jazlah has prescribed a mixture of sweet PJ with honey for earache and sour one for treating pterygium (15). Ibn Beytar and Tonekaboni believed that the administration of eardrop prepared by mixing pomegranate with rose oil (*Rosa damascene*) would heal the earache (14, 75).

ITM scientists did benefit from pomegranate for the treatment of eye problems. Antaki has mentioned: “Concentrated PJ (by the sun or by heating in a copper pan) sharpens the eyesight and is useful for the treatment of epiphora and pannus” (12). Some ancient scholars believed that eating three pomegranate calyxes protects the eye from conjunctivitis for up to a year. If the sweet PJ is put in a glass container (Gharooreh) in front of the sun to make it thick and then applied in the form of kohl (a traditional kind of mascara), it would improve the vision; the older the mixture becomes, the better it yields result (19).

*Evidence from modern medicine (Eyes, ears, and nose)*

Both *in vitro* and *in vivo* studies on *P. granatum* have indicated the protective effect of multiple preparations of this plant on retinal cell injury and ototoxicity (Table 7). However, the scope and variety of studies on this aspect are limited and clinical trials are lacking. Thus, further studies on pomegranate activities, particularly its anti-oxidant properties that have the ability to diminish or reverse age-related ocular degeneration, are needed.

*Metabolic disorders*

In ITM textbooks, it has been widely emphasized that pomegranates are useful for the treatment of metabolic diseases (13, 75). It is mentioned in many books that PJ imparts a rosy-colored appearance to the face (12, 13, 75), implying its beneficial effects on the metabolic system. The effects of pomegranate on metabolic disorders can be tracked in two main organs discussed in the following paragraphs. In our previous publication, the beneficial effects of pomegranate on different components of metabolic disorders were thoroughly discussed (9).

*Liver*

ITM scientists claim that pomegranate is a cooling agent for the liver (12, 14, 74) so it can decrease the extra heat in this organ in pathogenic conditions (14). For instance, PJ has been recommended for removing the harmful effects of alcohol from the liver. Pomegranate is claimed to act as an antidote for alcohol-induced hepatotoxicity (14, 19). Many ITM researchers have mentioned that pomegranate can remove the heat in the liver caused by eating too much wine (19). However, pomegranates should be used with caution in people with cold temperaments (14). In addition, all types of pomegranate have been prescribed for treating jaundice (12).

*Evidence from modern medicine*

Studies (*in vitro* and *in vivo*) have revealed that pomegranate extracts from the peels, flowers, juice, and seeds can control lipid metabolism in metabolic disorder-related illnesses like atherosclerosis, nonalcoholic fatty liver disease, and type 2 diabetes, preventing the onset of these conditions (9, 169). For instance, in high-fat-fed rats, pomegranate vinegar may control lipid metabolism and lessen liver damage (170).

The beneficial activities of pomegranate extracts and preparations have been evaluated in a range of liver-related disorders. As an example, an alcoholic extract from the flowers of *P. granatum* has been found to abrogate ferric nitrilotriacetate-induced hepatotoxicity in mice (171). PPE may stop liver fibrosis in biliary-obstructed rats by reducing oxidative stress or increasing endogenous anti-oxidant levels (172). Pomegranate extract has also shown protective effects such as lowering triglyceride and cholesterol content of cells, normalizing the expression of pro-inflammatory cytokines, and improving mitochondrial complex activity in obesity-associated nonalcoholic fatty liver disease (1).

In addition, different nanoformulations have been engineered for improving the activity of pomegranate on liver disorders. For example, silver nanoparticles biosynthesized using *P. granatum* leaves have shown improved antidiabetic potential *in vitro* (173). Through boosting fatty acid consumption in hepatocytes, pomegranate-derived omega-5 nanoemulsion might reduce hepatic steatosis in mice fed a high-fat diet (174).

*Heart and cardiovascular system*

In ITM, pomegranate (especially sour ones) has been described as a hematopoietic (14, 19), anti-palpitation (14, 15, 17, 18, 75), and vasodilatory agent (12, 17, 19, 74). Avicenna in his book Cardiac Medications: “All types of pomegranates including, sour, sweet, and sour-sweet ones are useful for the treatment of palpitation”. It is written in Al-Hawi and many other ITM textbooks that sour pomegranate acts like a polish for the heart and has the ability to burnish the cardiovascular system (14-16, 19, 76); accordingly, it can be deduced that pomegranate has anti-atherosclerosis activity (17, 74-76). Sour pomegranate prevents the flow of waste materials to the body (15) and quenches the excitation of yellow bile, black bile, and blood humor (14, 74, 75). Razi in Al-Hawi has mentioned: The pomegranate paste particularly the one concentrated in a copper pan is used to remove the excess amount of these humors (19).

*Evidence from modern medicine*

Pomegranate and its derivatives have been found to have protective effects on the cardiovascular system, according to several *in vivo* and clinical investigations. These vasculoprotective actions include lowering oxidative stress, improving macrophage, endothelial cell, and platelet function, decreasing blood glucose levels, lowering lipid oxidation, and having vasodilatory effects in addition to lowering blood pressure by inhibiting ACE activity (175). For instance, *P. granatum* flower extract could diminish cardiac fibrosis in Zucker diabetic fatty rats by modulation of cardiac endothelin-1 and NF-κB pathways (176). It could also protect isoproterenol-treated rats against myocardial damage by acting as a free radical scavenger and conserving the endogenous anti-oxidant system (177). A study showed how pomegranate, prickly pear, and apple juice vinegar can protect against swelling, hypertrophy, and fibrosis in obesity-related heart damage (178). Moreover, in individuals with unstable angina, PJ may lessen the frequency, onset, and length of angina pectoris attacks (179). In addition, according to the literature, pomegranate has the ability to reduce blood pressure. For example, the data from a meta-analysis evaluating 8 RCTs showed that PJ can significantly decrease both systolic and diastolic blood pressure levels (180).

***Other traditional systems of medicine around the world***

Table 8 shows the medicinal application of pomegranate in different countries around the world. Most of the usages mentioned in the table are similar to the prescriptions in ITM textbooks showing the knowledge transfer from Persia to other parts of the world. Similarly, different preparations from all parts of pomegranate including leaves, seeds, fruits, and peels are mentioned. Pomegranate is a well-known medicinal plant in Indian traditional systems of medicine including Ayurveda and Unani medicine.

**Table 1:** Some of the main phytochemicals reported from *Punica granatum*

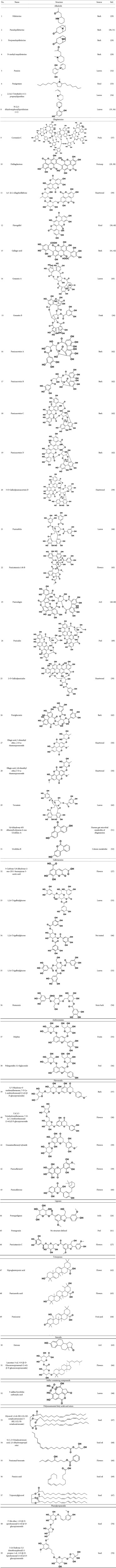

Male unproductive blossoms (left) vs female productive blossoms (right)(©Mesarbustes.fr)

**Table 2:** Therapeutic potential of *Punica granatum* on the gastrointestinal system

**Model**	**Activity**	**Plant part/compd.**	**Study design**	**Mechanism**	**Ref.**
Bacterial	Antibacterial properties against enteropathogens	Fruit exocarp (aqueous and methanol extracts)	Antibacterial activity against two *E. coli* species, two *Shigella sonnei *species, two *Shigella flexneri* species, and two *Salmonella* species	Both extracts were active against all microorganisms with inhibition percentage ranging from 36 to 100%; MIC values ranging from 1 to 4 mg/ml	(77)
Bacterial	Antibacterial activity against enterohaemorrhagic *E. coli*	Fruit shell (aqueous extract)	*E. coli *O157:H7, O26:H11, O111:NM and O22; Paper disc agar diffusion method	Growth inhibition; MIC= 0.19mg/ml and MBC= 0.39 mg/ml	(82)
Bacterial	Antibacterial activity against *H. pylori*	PPE (methanol extract)	Disc-diffusion method; *H*.* pylori *strains; 100 µg/disc plant extract, standard = 8 µg/disc Metronidazole	Growth inhibition; Inhibition zone diameter= 39±3.4 mm	(83)
Bacterial	Antibacterial effect against *H. pylori*	PPE ethanol extract	Twelve *H. pylori* clinical isolates; Disk diffusion method; Two-fold agar dilution method	↓Urease activity; IC_50_= 6 mg/ml	(84)
Protozoal	Antiprotozoal activity	Fruit exocarp (methanol extract)	Susceptibility assay; Subculture method; *Entamoeba histolytica* strain HM1-IMSS and *Giardia lamblia* isolated IMSS:1090:1; Concentrations (2.5–200 µg/ml); Positive controls=metronidazole and emetine, control=culture medium plus trophozoites and DMSO, blank=culture medium	Active against *Entamoeba histolytica*; IC_50_= 29.5 µg/ml	(85)
Cellular	Chemopreventive against colon cancer	Urolithin-A	*In vitro* (colon cancer cells), and *ex vivo* in adenoma and normal mucosa from Pirc rats	↓COX-2 protein expression; ↑C-CASP-3 expression	(86)
Cellular	Antiproliferative in colon cancer cells	PJ	Human colon carcinoma cell lines HT-29	↑miR-126; ↓VEGF, IGF and pPI3K, AKT	(87)
Cellular	Anti-inflammatory effect	Pomegranate beverage (polyphenolics)	Lipopolysaccharide (LPS)-treated CCD-18Co colon-myofibroblastic cells	↓Ki-67 proliferative index and miR-145; ↓TNF-α, IL-1β, COX-2, and iNOS, p70S6K1 and HIF1α	(88)
Cellular	Suppressing cancer progression and tumor angiogenesis of pancreatic and colon cancer	Pomegranate fruit extract	Human pancreatic cancer (Suit-2) and colon (colo205); Chick chorioallantoic membrane (CAM) cancer implant model	↓Tumor weight and Hb concentrations, FGF2 expression	(89)
Animal	Anti-cancer effect	PPE (ethanol extract)	HPLC for determining EA in samples. MTT assay, apoptosis and scratch assay, gelatin zymography, and quantitative RT-PCR to determine the anti-cancer properties, immunosuppressed C57BL/6 mice carrying human gastric adenocarcinoma cell line	↓P53, BAX, APAF1, BCL2, iNOS, NF-κB, IL-8 and TNF-α; ↑Cancer cell death	(90)
Animal	Antidiarrheal effect	PPE (aqueous extract; ethyl acetate fraction)	BALB/c mice and Wister rats; orally; 100, 200, 400 mg/kg; 4 hr; *in vivo *castor oil-induced diarrhea and *ex vivo* ileum tissues	↓Diarrhea by inhibiting gastrointestinal transmission and intestinal juice accumulation; Protects against intestinal epithelium injury induced by castor oil	(78)
Animal	Antidiarrheal effect	PPE (aqueous extract)	Adult albino rats; Intraperitoneal injection; 100, 200, 300, and 400 mg/kg; Standard=loperamide; 1 ml of castor oil orally; 5 hr	↓Diarrhea in a dose-dependent manner by inhibiting intestinal motility and intestinal fluid accumulation; IC_50_= 174±4 mg/kg	(91)
Animal	Anti-inflammatory activity on the gastrointestinal tract	PPE (aqueous extract)	*In vitro* (Caco-2 cells) and *ex vivo* (porcine colonic tissue explants)	↓Pro-inflammatory cytokines (IL-1A, IL-6 and CXCL8)	(92)
Animal	Antisecretory activity on cholera toxin-induced intestinal secretion	Fruit exocarp (methanol and aqueous extract)	Rat jejunal loops model; Male Sprague–Dawley rats; 300 mg/kg orally; Standard=loperamide	Inhibition of intestinal secretion by 55.9±3.6% in methanol and 19.1±6.9 % in aqueous extract	(93)
Animal	Inhibition of gastric mucosal injury	Fruit rind (methanol extract)	Male Wistar rats; 250 mg/kg and 500 mg/kg plant extract; Standard=ranitidine 400 mg/kg; Aspirin- and ethanol-induced gastric ulceration	↓Lipid peroxidation levels; ↑Glutathione levels; Inhibition of aspirin-induced gastric ulcer (22.37-74.21%) Inhibition of EtOH-induced gastric ulcer by 21.95 and 63.41%	(94)
Animal	Reduction in inflammation and ulceration scores in intestinal colitis	Pomegranate beverage (polyphenolics)	Dextran sodium sulfate (DSS)-induced colitis in Sprague–Dawley rats	Protection against DSS-induced colon inflammation and ulceration (50% and 66.7%, *P*=0.05 and 0.045, respectively)	(88)
Animal	Inhibition of diarrhea and gastrointestinal transit (GIT)	Fruits (Gabsi and Garsi varieties); (Juice, total extract, and methanol extract)	Male Wistar rats; Castor oil-induced diarrhea and charcoal meal test (10% charcoal in 5% gum Arabic); Standard antidiarrheal drug=diaretyl (10 mg/kg, p.o.)	↓Diarrhea dose-dependently and GIT dose-dependently; The Garsi variety was more effective	(95)
Animal	Chemopreventive against colorectal cancer	Mesocarp (decoction)	Pirc rats; Orally; 6 weeks	↓Mucin-depleted foci, as CRC biomarkers (*P*=0.02); ↑Apoptosis (*P*<0.01)	(86)
Animal	Protective effect in Crohn’s disease	Ellagic acid	Male Wistar rats; Induction of colitis by trinitrobenzene sulfonic acid (TNBS); Ellagic acid (10–20 mg/kg p.o. administered by gavage 48, 24, and 1 hr prior to the induction of colitis and 24 hr later	↑Mucus production in goblet cells; ↓Neutrophil infiltration and COX-2 and iNOS overexpression, activation of p38, JNK, and ERK1/2 MAPKs	(96)
Animal	Antioxidant against azoxymethane-induced colon cancer	PPE	Male Sprague-Dawley rats; Chow diet and normal tap water; 2 intraperitoneal injections of AOM dissolved in physiological saline once a week (15 mg/kg) for 2 weeks	↑Glutathione and TAC levels in colonic mucosal tissue	(97)
Animal	Suppressing azoxymethane-induced colorectal aberrant crypt foci and inflammation	PJ	Male Sprague-Dawley rats, received PJ (2504.74 mg gallic acid equivalents/l), intraperitoneal injection of AOM 15 mg/kg for 2 weeks	↑miR-126; ↓COX-2, iNOS, NF-κB (p65) and VCAM-1, IGF and PI3K, AKT, mTOR mRNA, and protein level	(87)
Animal	Anti-inflammatory activity in the acute and chronic colitis	Ellagic acid	Female BALB/c and C57BL/6 mice; Dextran sulfate sodium-induced colitis; 100 mg/d/mouse ellagic acid administered orally	↓COX-2, iNOS, NF-κB, IL-6, TNF-α, and IFN-γ	(98)
Animal	Reducing colitis-induced visceral pain	Pomegranate mesocarp decoction; Polysaccharides; Ellagitannins	Male Sprague–Dawley rats; 2,4-dinitrobenzenesulfonic acid-induced colitis; Pomegranate whole decoction (300 mg/kg), polysaccharides (300 mg/kg), and ellagitannins (45 mg/kg) orally for 14 days	↓Mast cells and density of collagen fibers in the mucosal stroma	(99)
Animal	Antibacterial effect against *H. pylori*	PPE (ethanol extracts)	Female Wistar rats, inoculated by gavage 1 ml/rat with *H. pylori *suspension of 9 McFarland twice daily, treated after 7 weeks with PPE 50 mg/kg	↓Urease activity	(84)
Clinical	Changes in gene expression in colon tissues from colorectal cancer patients	Pomegranate extract	RCT; Programmed colonoscopy (n=2501), older than 18 years and confirmed CRC diagnosis	↓CDKN1A, EGFR, TYMs, CD44, CTNNB1	(100)

^1^ Pomegranate peel extract; ^2^ Pomegranate juice

**Table 3:** Therapeutic potential of *Punica granatum* on the respiratory system

**Model**	**Activity**	**Plant part/Compd.**	**Study design**	**Mechanism**	**Ref.**
Simulation	Antiviral activity against SARS-Cov-2	PPE; Punicalagin; Punicalin; Urolithin A	Molecular docking simulation through Yasara Structure software based on the AutoDockLGA algorithm and AMBER03 force field; Molecular dynamics simulation in LARMD server	↓SARS-CoV-2 S-glycoprotein binding ability to ACE2 receptor PPE MIC= 0.06 mg/ml Punicalin MIC= 0.14 mg/ml	(102)
Bacterial	Antimicrobial activity against *Mycobacterium tuberculosis* and b-lactamase-producing *Klebsiella pneumoniae*	Fruit compounds: caffeic acid and ellagic acid; EGCG and quercetin	Double-disc synergy; Phenotypic confirmatory test for ESBL detection; Modified Hodge test	PPE: antimycobacterial activity; MIC 64–1024 mg/ml	(103)
				PJ: antimycobacterial activity; MIC 256 -41024 mg/ml	
				EGCG and quercetin: antitubercular and antibacterial; MIC 32–256 mg/ml; MIC 64–56 mg/ml	
				Caffeic acid and ellagic acid: antitubercular and antibacterial activity; MIC 64–512 mg/ml	
Viral	Antiviral activities against human respiratory syncytial virus (RSV)	Fruit cortex (aqueous extract)	Cytopathic effect reduction assay	Anti-RSV activity IC50= 62.5 µg/ml	(104)
Viral	Antiviral activity against SARS-Cov-2	PPE	ABTS assay for antioxidant effects; SARS-CoV2 inhibitor screening assay kit; Three extract concentrations ranging from 0.04 mg/ml to 1 mg/ml; 3CL protease assay (0.04 and 0.2 mg/ml)	↓SARS-CoV-2 S-glycoprotein binding ability to ACE2 receptor and Activity of the virus 3CL protease	(20)
Viral	Antiviral activity against SARS-Cov-2	Punicalagin	3CL protease assay kit (BPS Bioscience)	↓Activity of the virus 3CL protease; IC50= 6.192 μg/ml	(105)
Cellular	Reduction in lung inflammation	PPE (Aqueous extract)	*In* *vitro*: human neutrophils	↓LPS-induced lung inflammation and myeloperoxidase activity	(106)
Cellular	Non-small cell lung carcinoma treatment	Leaves extract	Non-small cell lung carcinoma cell line A549, H1299 and mouse Lewis lung carcinoma cell line LL/2; Cell viability and colony formation assay; Wound-healing migration assay; Cell cycle and apoptosis analysis by flow cytometry; Mitochondrial membrane potential (∆Ym) assay; Detection of ROS	Arresting cell cycle progression in G2/M; Blocked H1299 cell migration and invasion; ↓Metalloproteinase 2 and 9 expressions, ROS and ∆Ym; IC50= 47 μg/ml	(107)
Cellular	Induced cell cycle arrest and apoptosis in human lung Adeno carcinoma A549 Cells	PJ	A549 cells treated with PJ (2% (v/v)) at several time exposures (0–72 hr); Quantification of apoptosis by Annexing V Labeling; Caspase-3, -8 and -9 analyses; MMP analyses	Induced cell cycle arrest at G0/G1 and apoptosis through intrinsic pathway; Loss of MMP; Release of cytochrome c in the cytosol; Activation of caspase-3 and -9	(108)
Cellular	Suppress microsomal prostaglandin E synthase-1 expression and induce apoptosis lung cancer	Ellagitannins; Leaves extract	A549 cells were incubated either with or without 10 ng/ ml IL-1β; 10 μM granatin A, granatin B, or geraniin for 24 hr; Enzyme immunoassay; TUNEL assay	↓mPGES-1 expression without affecting COX-2, TNF-α, inducible nitric oxide synthase, anti-apoptotic factor; B-cell chronic lymphocytic leukemia/lymphoma 2	(43)
Cellular	Anticancer activity	Fruit extracts of immature pomegranate	Human lung H1299 adenocarcinoma cells; 100 μg/ml of extracts from different fruit matrices	Induction of caspase-3 activity	(109)
Animal	Reduction in lung inflammation	PPE (Aqueous extract)	Male BalbC mice; intraperitoneal injection of 200 mg/kg extract; LPS-induced lung inflammation (5 µg intratracheal LPS instillation)	↓LPS-induced lung inflammation and myeloperoxidase activity	(106)
Animal	Alleviating asthma	Leaves (ethanol extract/microencapsulated extract)	Female BALB/c mice; Ovalbumin-induced asthma; Encapsulated extract (10 mg/ml, 25 µl per nostril)/ non-encapsulated pomegranate extract (20 mg/Kg, 25 µl per nostril); Intranasal instillation; Standard=dexamethasone	↓Leukocytes' (eosinophils) recruitment to bronchoalveolar fluid, IL-1β, and IL-5 in the lungs	(110)
Animal	Bronchospasmolytic effects	PPE (Aqueous extracts)	Isolated guinea pig trachea chains; Contractions induced by acetylcholine or histamine; 10 mg/ml plant extract	↓Force of contraction by histamine by 30-70%	(111)
Animal	Protection against acute lung injury	Alloyl-hexahydroxydiphenoyl (HHDP)-glucose (isolated from leaves)	Male BALB/c mice; intra-tracheal lipopolysaccharide (LPS)-induced acute lung injury; galloyl-HHDP-glucose (5, 50, and 100 mg/Kg); standard= dexamethasone at 5 mg/Kg	↓TNF-α, IL-6, and IL-1β gene expression and protein levels, lung inflammation, and cell accumulation	(112)
Animal	Protection against acute lung injury	Punicalagin	RAW 264.7 murine macrophage cell line; Immunocytochemical analysis	↓TNF-α, IL-1β, IL-6, protein concentration and myeloperoxidase activity; Suppressing p38 MAPKs and NF-κB pathways	(113)
Animal	Protection against acute lung injury	Punicalagin	Male BALB/c mice with acute respiratory distress syndrome induced by intranasal instillation of LPS (20 mg/kg); Treated with punicalagin (12.5, 25, and 50 mg/kg) 1 hr prior to LPS exposure; Control=dexamethasone (5 mg/kg)	↓TNF-α, IL-1β, IL-6, macrophage and neutrophil infiltration, myeloperoxidase activity, TLR4 expression and NF-κB activation pathways	(114)

^1^ Pomegranate peel extract; ^2^ Epigallocatechin Gallate; ^3^ Pomegranate juice

**Table 4:** Therapeutic potential of *Punica granatum* on skin and wound

**Model**	**Activity**	**Plant part/compd.**	**Study design**	**Mechanism**	**Ref.**
Bacterial	Antimicrobial and antivirulence activities against *Pseudomonas aeruginosa*	PPE (Aqueous extract)	*P. aeruginosa* collected from burn wound cultures	↓Bacterial gelatinase activity by 40.28±2.35%; ↓Lecithinase activity by 53.84±4.89%; MIC= 1.4 mg/ml	(118)
Cellular	Protection against skin photoaging induced by UVB irradiation	Rind, seed, and fruit (methanol extracts)	Normal human dermal fibroblasts; Irradiated with UVB (170 mJ/cm2) for 1 min.	↑Procollagen type I and MMP-1 expression (especially by rind extract); ↑Collagen synthesis	(119)
Cellular	Inhibition of melanin content	Fruit (ethanol extract)	Melan-a melanocyte cells; Melanin inhibition assay; Fruit extract standardized to 20% punicalagin; at concentrations of 50 µg/ml and 100 µg/ml	↓Melanin content by 40% (50 µg/ml) and 60% (100 µg/ml)	(120)
Cellular	Anti-tyrosinase activity	Rind (methanol extract)	Murine melanoma B16 F0 cells; Positive control=Kojic acid (1%); Extract concentrations (200–500 mg/ml)	Tyrosinase inhibition at 500 μg/ml= 82.3±5%	(121)
Cellular	Inhibition of melanin production	Fruit (ethanol extract)	Testing* Salvia hispanica* (chia) seed extract and *P. granatum* fruit extract (20% punicalagin), in combination or alone; Melanin inhibition assay: Melan-a cells, positive control=phenylthiourea 60 μg/ml; Tyrosinase enzyme assay: enzyme extracted from B16 melanoma cells, positive control=kojic acid; Melanogenesis-related gene expression (RT-PCR) analysis: Melan-a cells, positive control=ascorbic acid-2-glucoside	↓Melanin content (1:1 ratio of the combination of extracts showed maximum efficacy); Not effectively inhibiting tyrosinase activity; ↓Tyrosine, Tyrp1 and Mc1r expression	(122)
Cellular	Protection against oxidative stress	Pomegranate extract	Human keratinocyte HaCaT cells treated with extract for 24 hr; Methylglyoxal-induced cytotoxicity,	↓Methylglyoxal-DNA adducts, reactive oxygen species; ↑Cell adhesion, migration, and wound healing capacity	(123)
Animal	Chemopreventive agent against skin cancer	Seed oil	Female CD1 mice; Skin cancer initiated with topical exposure of 7,12-dimethylbenzanthracene and with biweekly promotion using 12-O-tetradecanoylphorbol-13-acetate (TPA); Pretreated with 5% pomegranate seed oil	↓Tumor incidence (*P*=0.05) and multiplicity; ↓TPA-induced ornithine decarboxylase (ODC) activity (17% reduction)	(124)
Animal	Cutaneous wound healing	Pomegranate peel polyphenols (PPP) gel	Adult alloxan-induced diabetic Wistar rats; Polyphenol mass fraction in PPP gel=30%;	↓Wound closure; ↑Fibroblast infiltration, collagen regeneration, vascularization, and epithelialization in the wound area; ↑Hydroxyproline, NO production, and NO synthase activities; ↑Transforming growth factor-β1 (TGF-β1), vascular endothelial growth factor (VEGF), and epidermal growth factor (EGF) expressions	(119)
Animal	Wound healing activity	PPE (methanol extract)	Wistar rats; Water-soluble gel of extract; Negative control=no gel, positive control=blank gel, commercial preparation=silver sulfadiazine; Treatment with 2.5% and 5.0% gel	↑Wound healing by 55.8% at 2.5% and 59.5% at 5.0% (on day 15); ↑Hydroxyproline content up to 34.5% (2.5%), and 48.5% (5.0%) compared with the negative control; ↑Hydroxyproline 24.1% (2.5%) and 38.0% (5.0%) compared with the positive control	(125)
Animal	Wound healing activity	Male abortive flowers (ethanol extract)	Male Wistar rats; reference standard (nitrofurazone ointment); topical treatment (extract ointment 200 mg/kg/day); Control (simple ointment); 16 days	↓Wound size, inflammation cells, collagen fibers, and re-epithelization	(126)
Animal	Anti-inflammatory activity on the skin	Rind (aqueous extract); Total pomegranate tannins (TPT)	Porcine skin mounted in Franz diffusion cells; Test solutions (1 ml): pomegranate rind extract (PRE) (consisted of 20% w/w punicalagin), PRE + ZnSO4, ZnSO_4_, TPT, tannin-free fraction (TFF) and control=phthalate buffer pH 4.5; Analysis of COX-2 by SDS PAGE and western blotting and Immunohistochemical (IHC)	TPT and PRE and PRE + ZnSO_4_: ↓COX-2 expression (after 6 hr and up to 24 hr); No anti-inflammatory activity by ZnSO_4 _and TFF and control	(127)
Animal	Antiaging effect on UVB-induced skin	Pomegranate juice concentrated powder (PCP)	Female SKH‑1 hairless mice; 6 groups: Intact vehicle control‑unexposed; UVB control‑UVB exposed (mice received vehicle control and UVB exposure); PCS 2 ml/kg ‑UVB exposed; PCP 100 mg/kg‑UVB exposed; PCP 200 mg/kg‑UVB exposed; and PCP 400 mg/kg‑UVB exposed; UVB irradiation three times a week for 15 weeks at 0.18 J/cm^2^; PCS (contained 2.31 mg/g ellagic acid)	Anti-apoptotic effects; Skin matrix metalloproteinase activity inhibition; ↓Wrinkles induced by photoaging; ↑Skin water contents, collagen type I, and hyaluronan contents; ↓IL‑1β; Inhibition of glutathione depletion	(128)
Animal	Wound healing activity	Whole fruit extract standardized with 40% ellagic acid	Male albino rats (*Rattus norvegicus*); Treatment of incised wounds with topical ointment of pomegranate extract at 2.5, 5, and 7.5% concentrations or Betadine® ointment, twice a day for 7 and 14 days	↑Wound healing in the application of 7.5% pomegranate extract ointment for 14 days; ↑Collagen deposition; ↓Polymorphonuclear neutrophils infiltration in wound area; ↑Angiogenesis	(129)
Animal	Wound healing activity	PPE	Inbred Sprague–Dawley male rats; excision wound model; Control group: petroleum jelly, experimental group: PPE (100 mg/kg) +petroleum jelly, standard group: mupirocin ointment (100 mg/kg); 15 days	Wound contraction of 95% (*P*<0.01) by day 15 in the experimental group; ↑Hydroxyproline content (*P*<0.05)	(130)
Animal	Anti-psoriasis activity	Punicalagin	Male BALB/c mice (6–8 weeks of age), imiquimod (IMQ)-induced psoriasis; Five groups: Control group, IMQ group, IMQ + DEX group (DEX, dexamethasone cream, positive drug for psoriasis), IMQ + Vehicle group, IMQ + PUN group (25 mg/kg PUN)	Inhibiting NF-κB-mediated IL-1β transcription and caspase-1-regulated IL-1β secretion	(131)
Clinical	Anti-aging activity	Arils (methanol extract)	12 human volunteers aged from 35 to 60 y/o, divided into 2 groups, 2 times daily topical application for a month, one group received a placebo	↓Free radicals	(132)
Clinical	Anti-aging activity	Fermented pomegranate extract	Double-blind, 35–55y/o, 40 healthy subjects, half on placebo, drink 50 ml daily; Another 40 subjects, half on placebo, apply 3 ml serum over their face 2 times daily for 4 weeks	↓Free radicals; ↑Skin moisture, brightness, and elasticity	(133)
Clinical	Skin lightening activity	PPE	Split-face, randomized, double-blind, placebo-controlled test of 30 subjects divided into 2 groups; Applied the preparation on half of their face	↓Free radicals; Tyrosinase and TRP-2 inhibition	(134)
Clinical	Protection against radiation-induced skin and mucosal changes	Whole fruit extract	Prospective, clinical, double-blind, case-control study; 60 patients (30 cases and 30 controls) undergoing radiotherapy for head and neck cancer; 12 months; 2 capsules/day for 6–7 weeks; each capsule containing 300 mg extract (40% polyphenols and 27% punicalagin)	↓Severity of radiation-induced mucositis and dermatitis (*P*<0.0001)	(135)
Clinical	Prevention or improvement of skin changes associated with striae	Seed oil	An interventional, nonrandomized study; 20 healthy females; 21–48 years old: 10 with striae albae at the hip level and 10 with no stretchmarks; Measurements of skin hydration level, skin elasticity, and thickness of the dermis at the beginning and after 3 and 6 weeks; Application of oil-in-water cream containing plants once daily	↑Dermis thickness, hydration, and elasticity values.	(136)
Clinical	Reduce photodamage from UVB irradiation	POMx® (Pomegranate extract); PJ	A parallel, three-arm, open-label RCT; n=72, female, 30–45 years, with Fitzpatrick skin type II-IV, 3 groups: 1–1000 mg of pomegranate extract (PomX), 2- oz of PJ, 3- placebo; 12 weeks; determination of minimal erythema dose (MED) and melanin index by cutometer;	↑MED; ↓Melanin formation	(137)
Clinical	Improvements in several biophysical properties, wrinkles, and shifts in the skin microbiome	Pomella® (Pomegranate extract)	Prospective, double-blind placebo-controlled study, healthy males and females aged 25–55 years; 4 weeks; BTBP 3D Clarity Pro® wrinkle assessment, SkinColorCatch® facial erythema index, and melanin index assessment	↓Wrinkle severity (*P*<0.01), forehead sebum excretion rate (*P*=0.14), facial transepidermal water loss (*P*<0.05)	(138)

**Table 5:** Therapeutic potential of *Punica granatum* on the reproductive system

**Model**	**Activity**	**Plant part/compd.**	**Study design**	**Mechanism**	**Ref.**
Viral	Antiviral activity against HSV 1 and 2	Freeze‑dried juice powder (FP); Aqueous extract (AE); Peels powder (PP)	HSV‑1 clinical strain and HSV‑2 ATCC G strain; Control=acyclovir; Cytopathic effect inhibition assay; MTT assay	Anti-HSV activity; Anti HSV 1 IC50: FP= 30.6 µg/ml; AE= 15.8 µg/ml; PP= 250 µg/ml; Anti HSV 2 IC50: FP= 18.14 µg/ml; AE= 17.6 µg/ml; PP= 185 µg/ml	(141)
Cellular	Anti-Ovarian cancer	Punicalagin	Human A2780 ovarian cells; Cell Counting Kit-8 assay; Flow cytometry analysis; Protein expression measured by western blot analysis; Wound healing assay	Arresting G1/S phase transition; ↑BAX, TIMP-2 and -3; ↓β-Catenin signaling pathway; activity, Bcl-2, invasion capability and activities of MMP-2 and MMP-9	(142)
Animal	Improvement of sperm quality	PJ	28 adult male Wistar rats, divided into 4 groups; Group 1: 1 ml distilled water (control), group 2: 0.25 ml PJ plus 0.75 ml distilled water, group 3: 0.50 ml PJ plus 0.50 ml distilled water, group 4: 1 ml PJ, daily for 7 weeks	↑Spermatogenic cell density, epididymal sperm concentration, and sperm motility; ↓ROS	(140)
Animal	Reverses the deleterious effect produced by lead acetate (LA)	Pericarp ethanol extract	30 adult male Holtzman rats; 5 groups: distilled water (control group), LA (lead acetate), LA with EEP (ethanol extract of pomegranate), LA with ascorbic acid (positive control), and EEP alone, respectively; 35 days	Protects the stages of mitosis (stages IX–XI), meiosis (stage XIV), and spermiation (stage VIII) in the spermatogenic cycle	(143)
Animal	Increases sex hormones	PPE (methanol extract); PJ	18 adult male albino rats; 3 groups: control, 200 mg/kg PPE, 3 ml/kg juice, administered for 21 days; Testis indexed after	↑Testosterone, FSH, LH, endogenous testicular antioxidant enzymes; ↓Lipid peroxidation and nitric oxide formation in testes	(139)
Animal	Protection of testes against carbon tetrachloride intoxication	PJ	28 Wistar albino male rats; 4 groups: control, CCl_4_, PJ and PJ+CCl_4_, CCl_4_ (2 ml/kg) administered via the intraperitoneal route once a week for ten weeks; PJ via drinking water 2 weeks before and concurrent with CCl_4_	↑Testosterone, FSH, LH, endogenous testicular antioxidant enzymes ↓Lipid peroxidation and nitric oxide formation in testes	(144)
Animal	Sperm improvement in testicular torsion-detorsion	PJ	21 male Wistar albino rats, 3 groups, control, ischemia/reperfusion (I/R), and PJ+I/R group (0.4 ml/day PJ orally over a period of eight weeks prior to surgery)	↑Spermatid, spermatocyte and spermatogonia concentrations; ↓Superoxide dismutase and malondialdehyde	(145)
Animal	Protection against 3G radiation-induced reproductive toxicity	PJ	Adult male Wistar rats, 5 groups, control, sham-exposed, 3G exposed, 3G exposed + juice and only juice groups, 3G exposure: 2 hr/day for 45 days, 6 days a week, vector signal generator model (VSG25A)	↑Sperm count, motility, and viability; ↓Oxidative parameters	(146)
Clinical	Improvement of erectile dysfunction	PJ	Double-blind RCT; Sixty sexually active, healthy males aged 21–70 years with a history of ED for at least 3 months duration, ED Score of 17–25 on the IIEF questionnaire, drink 8 ounces of juice every day for 28 days	↑NO activity in vascular endothelial cells	(147)
Clinical	Anti-hemorrhagic activity against heavy menstrual bleeding	Flower	Double-blind RCT; In comparison with oral tranexamic acid, 123 eligible patients (20 to 49 years), non-anemic (hemoglobin (Hb) of≥10.5g/dl), had no history of previous thromboembolic disorders, chronic illnesses, or other diseases known to interfere with menstrual bleeding	↓Duration of bleeding and menstrual blood loss	(148)

**Table 6:** Therapeutic potential of *Punica granatum* on mouth and teeth

**Model**	**Activity**	**Plant part/compd.**	**Study design**	**Mechanism**	**Ref.**
Bacterial	Antimicrobial activity against *Streptococcus mutans*	Pericarp extract	Disc inhibition zone method and broth dilution assay; Control=chlorhexidine 0.2%	Growth inhibition; MIC= 50 mg/ml	(153)
Bacterial	Antifungal efficacy on *Candida albicans*	PPE (aqueous decoction)	Disc diffusion method; The zones of microbial (cultures of *Candida albicans*) inhibition at 18 and 24 hr	Antifungal activity (*P*<0.01); Inhibition zone 18.8 mm after 24h and 22 mm after 48 h	(154)
Bacterial	Antimicrobial inhibition as a mouthwash	PPE (methanol extract)	Agar well method (n=3); Incubated at 37 °C for 24 hr; Repeated 3 times; Bacterial strains: *Staphylococcus mutants*, *Staphylococcus aureus*, *E. coli*, *Klebsiella pneumonia*, *Streptococcus gaseous, *and *Streptococcus faecalis*; 25%, 50%, and 75% methanol extract; Control=chlorhexidine gluconate mouthwash	The 50% and 75% extracts exhibited the highest inhibition on *S. mutants*	(155)
Bacterial	Antibacterial activity against dental caries and gingivitis *S. mutans*	PPE (hot and cold aqueous extracts)	Agar disk diffusion method; *S. mutans* was isolated from fifty-five patients; 10 μl of aqueous peels extract or 0.2% chlorhexidine (standard); incubated at 37 °C for 24 hr	Inhibition zones: hot (31 mm), cold (27 mm), chlorhexidine (25 mm)	(156)
Bacterial	Antibacterial activity against *Enterococcus faecalis* and *Candida albicans *in root canal dressing	PPE (ethanol extract)	Inocula of 5.0 × 105 CFU/ml *E. faecalis *and 1.0 × 103 CFU/ml of *C. albicans*	↓Cell viability, biofilm formation; *C. Albicans* MIC= 62.50 µg/ml; *E. faecalis *MIC= 15.62 µg/ml	(157)
Cellular	Anti-cancer activity against oral cancer	PPE (ethanol extract)	KB 3-1 oncogenic cell culture; MTT assay; Extract concentrations of 50, 100, 150, 200, 250, 300, and 350 µg	↓Cell viability	(158)
Animal	Antifungal activity against oral candidiasis	PPE (methanol extract)	Male Wistar rats immunosuppressed with cyclosporine (40 mg/kg/d) and hydrocortisone acetate (500 μg/kg/d) except the control group; Induction of oral candidiasis via the oral administration of *Candida albicans *on the palate and tongue; extract 125, 250, and 500 μg/ml/kg and nystatin 100000 U/ml/kg by gavage daily; 15 days	↓Growth of *C. albicans* 100% cure in all the doses after 15 days; Most effective Conc.: 500 μg/ml after 5 days	(159)
Clinical	Antimicrobial prophylactic activity on oral bacteria	Fresh fruit juice	20 patients; A group received 0.2% chlorhexidine mouthwash (10 ml), the other group was given freshly prepared PJ as a mouthwash (75 ml for 2 min); salivary sample pre-rinse and after 15 min	↓Colony-forming units by pomegranate mouthwash (51.1%) and chlorhexidine mouthwash (71.3%)	(160)
Clinical	Gingival bleeding reduction	Fruit (ethanol extract)	Double-blind, interventional, experimental, longitudinal, and prospective RCT, with an inductive approach; N= 55, 18-56 years old; Control=chlorhexidine 0.12% solution mouthwash; Study groups: pomegranate extract mouthwash (twice daily, 1 min long, with 10 ml of the solution); Ainamo and Bay gingival bleeding index (GBI) on 0, 7, and 15 days	↓Gingival bleeding index (*P*<0.001)	(161)
Clinical	Reduction of gingivitis risk	PomElla® (pomegranate extract)	Single-blinded RCT; 19–25 years old, n=32, split evenly among both genders; 4 weeks; saliva sample donation before and after the experiment; randomly assigned pomegranate extract PomElla® dissolved in water or placebo: cornstarch in water (three times a day for 1 min per rinse)	↓Total protein (*P*<0.01), aspartate aminotransferase activity (*P*<0.005), α-Glucosidase activity (*P*<0.05); ↑Antioxidant enzyme ceruloplasmin activities (*P*<0.05), radical scavenging capacity (*P*<0.05)	(162)
Clinical	Antibacterial activity against *S. mutans*	Fruit (mouthwash)	A single-blinded, parallel-group RCT; *n*=20; experimental group (I): *P. granatum* and controlled group (II): chlorhexidine mouthwash; plaque samples evaluated for *S. mutans* at baseline and 15th day	↓Mean plaque both groups (group I *P*=0.043 and group II *P*=0.047); No significant difference between the two groups at 7^th^ day	(163)
Clinical	Treatment of minor recurrent aphthous stomatitis	*P. granatum* flowers (pleniflora, sweet Alak, and Saveh black varieties) (ethanol and aqueous extracts)	Double-blind method; n=210 (69 F and 141 M); experimental group: alcoholic or water extracts (both 10% in 100 ml), negative control: received nothing; using mouthwash for 10 min, four times a day, for 10 days; measuring the size of lesions at days 1,2,4,6,8, and 10, The pain satisfactory degree was recorded	↓Entire-time of complete treatment; ↑Patients’ satisfactory	(164)
Clinical	Antimicrobial activity against *S. mutans*	Pericarp extract	RCT; 30 children, 6-12 years; Control=chlorhexidine 0.2%; Samples collected before and after mouth rinse	↓Salivary *S. mutans* count; No statistically significant difference between PPE and chlorhexidine	(153)

**Table 7:** Therapeutic potential of *Punica granatum* on eyes, ears, and nose

**Model**	**Activity**	**Plant part/compd.**	**Study design**	**Mechanism**	**Ref.**
Cellular	Protecting human retinal pigment epithelium (RPE) cells from photo-oxidative stress	Punicalagin	Human RPE cell line (ARPE-19); Exposed to UV-A radiation for 1, 3, and 5 hr; ARPE-19 cells were pre-treated with punicalagin (24 h); ROS, BAX, and BCL-2 detection	Activating Nrf2/HO-1 signaling pathway; ↓Apoptosis and BAX/BCL-2 ratio; Antagonizing the decrease in cell viability and reduced high levels of ROS	(165)
Cellular	Anti-cataract activity	Leaves (methanol extract)	Glucose-induced cataract model in goat lenses; Positive control=quercetin (500 μg/ml); Leaves extract concentrations: 250, 500, 1000 μg/ml); 72 hr	Aldose reductase inhibition; ↓Oxidative stress; ↑Antioxidant defense system; IC50= 83.55 ± 3.92 μg/ml	(166)
Animal	Protection on experimental ischemia/reperfusion (I/R) retinal injury	Pomegranate extract	Male albino rats; Groups I and II (sham-operated and received saline or extract, respectively), groups III and IV (I/R) rat models with prior administration of saline or 250 mg/kg/day extract, respectively	PMG prevented I/R-induced retinal damage; ↑Nuclear factor erythroid 2-related factor 2 (Nrf2) immunoreactivity; ↓NO	(167)
Animal	Protection against amikacin-induced ototoxicity	PPE (≥98% ellagic acid)	BALB/c mice; Control group: physiological saline (100 μl/day) via gavage; Amikacin (AMK) group: intraperitoneally received AMK intramuscular injection at 500 mg/kg/day for 15 consecutive days; PPE plus AMK group: hypodermic injection for AMK at 500 mg/kg/day for 15 consecutive days and PPE (34 mg/kg, 100 μl/day) via gavage for 5 days prior to AMK injection and for 15 days concomitantly with AMK injections; PPE group: PPE via gavage for 20 days; auditory brainstem response (ABR) was recorded 1 day before and 15 days after AMK treatment	Regulating the MAPK/FoxO3a signaling pathway in the cochlea; IC50= 45 μg/ml	(168)

**Table 8.** Application of pomegranate in other countries

**Country**	**Parts used**	**Mode of preparation/ administration**	**Uses**	**Ref.**
Algeria	Barks	Decoction, infusion, poultice, cooked	Colon ailments, wounds, stomach ulcers, diarrhea, cough	(181)
	Peels	Infusion, powder	Aphthae (as a mouthwash), diarrhea, and digestive problems	(149)
Brazil	Fruits, fruit peel, and seed	Decoction, maceration, syrup	Diarrhea, female infection, general infection, worms, cancer, myoma, depurative, diabetes, flu, stomach pain, gastritis, indigestion, ulcer, rheumatism, ovarian cyst, vaginal discharge, uterine infection, vaginal infection, uterine inflammation, wound healing, throat infection, throat inflammation	(182)
	Fruit, bark	Mouthwash, gargle	Acute respiratory infections in children; sore throat	(183)
India	Seeds	Botanical mixtures	Kidney and urinary disorders	(184)
	Leaves	Infusion	Cuts and wounds	(185)
	Whole fruit	Paste (external)	Snake bites, scabies, salivation with nausea, antifertility, skin diseases	(186)
Pakistan	Leaves/ fruit	Powder/ raw	Skin diseases, dysentery/ astringent, blood purifier, laxative, whooping cough, anthelmintic, diarrhea	(187)
	Fruits	-	Whooping cough	(188)
	Seeds	Powder/ oral	Abdominal worms of children	(189)
Palestine	Fruit/ peel	Squeeze and apply; mill the peel, soak in warm water, and apply	Hair and scalp treatments	(190)
Mexico	Pericarp	Syrup	Dental caries; gum diseases; aphthous ulcers; mouth sores	(191)
Morocco	Bark	Decoction	Diabetes, dermocosmetology, digestive system disorders, cardiac and renal diseases	(192)
Turkey	Fruit juice	Pressing/ oral	Diabetes	(193)
Yemen	Fruit/ peel	Decoction	Oral: Gastrointestinal ailments: stomach ulcer, diarrhea, ascariasis, tapeworm Respiratory tract diseases: asthma	(194)
		Paste	Dermal diseases: face pimples	

## Conclusion

Pomegranate fruits, leaves, blossoms, and seeds have all been used to cure several ailments in Persian and Islamic traditional medicines. However, pomegranate fruit juice and peel have received far more attention than the other parts. By comparing the ITM findings to modern medical evidence, we can deduce that a significant part of ITM findings is confirmed by modern medicine (195-197). For instance, the anti-bacterial activity of pomegranate in treating wounds, and infections such as respiratory, mouth, teeth, gastrointestinal, and uterine infections were well established in ITM books. Another instance of this conformity is the anti-oxidant property of pomegranate which is useful in the treatment of inflammation, skin aging, atherosclerosis, and metabolic disorders.

Though some of these traditional medical practices have been studied by modern medicine, many are still in their very early stages to be investigated. Although the main bioactive constituents of pomegranate preparations are polyphenols, there are yet insufficient data on the identification and isolation of the bioactive chemicals that are responsible for the biological activities. In addition, more preclinical and clinical research is demanded to confirm the other traditional applications, find the mechanisms of action, and evaluate the efficacy and safety. Overall, the potential of this valuable fruit and functional food can be fully realized via examination of the variety and interrelation of pomegranate phytochemicals as well as preclinical and clinical studies of their bioactivities.

## Authors’ Contributions

M A designed the study; Z B and M M collected data and drafted the manuscript; M A and M M discussed the results and strategy; SA E and M A supervised the study; M A and SA E approved of the final version to be published.

## Funding

This research did not receive any specific grant from funding agencies in the public, commercial, or not-for-profit sectors.

## Declaration of competing interest

The authors declare that they have no known competing financial interests or personal relationships that could have appeared to influence the work reported in this paper.
